# Factors Influencing Poor Implementation of Primary Preventive Care in Primary Healthcare Facilities of a Selected Municipality, Limpopo Province: Empirical Research Qualitative

**DOI:** 10.1002/nop2.70326

**Published:** 2025-09-28

**Authors:** Bongani Kwinika, Seani Adrinah Mulondo

**Affiliations:** ^1^ Department of Advanced Nursing Science University of Venda Thohoyandou South Africa

**Keywords:** facilities, factors, implementation, influencing, primary healthcare, primary preventive care, professional nurse

## Abstract

**Aim:**

To determine factors influencing poor implementation of primary preventive care in primary healthcare facilities of a selected municipality.

**Design:**

The study adopted a qualitative approach, an exploratory and descriptive design, to gain in‐depth data about the study topic.

**Methods:**

The study was conducted in five primary healthcare facilities in a selected municipality. A non‐probability purposive sampling approach was used to select fifteen (15) primary healthcare professional nurses to participate in the study. Data was collected for a period of 1 month using semi‐structured in‐depth face‐to‐face interviews. Data analysis was done following Tesch's eight steps to develop themes and subthemes. Trustworthiness was ensured through credibility, transferability, dependability and conformability. Ethical clearance certificates were obtained, and ethical standards were observed throughout the study.

**Results:**

The study findings indicated that the shortage of material and human resources, financial management‐related factors, personnel impacts, attitudes and beliefs of consumers towards services, support systems, and the increased burden of disease are factors influencing poor implementation of primary preventive care in primary healthcare facilities of a selected municipality. The study recommends that an increased budget for preventive care could curb the poor implementation of primary preventive care services in primary healthcare facilities.

**Impact:**

The study addressed the poor implementation of primary preventive health services in a selected municipality. Factors such as a shortage of material and human resources, financial management‐related factors, personnel impacts, attitudes and beliefs of consumers towards services, support systems, and an increased burden of disease were revealed as associated with the poor implementation of primary preventive care in primary healthcare facilities of a selected municipality. Policymakers should collaborate with community stakeholders and healthcare providers to review available health policies and programmes. The revisions of health policies could guide the health facilities to improve the implementation of PPC and realise universal health coverage through the National Health Insurance (NHI) for the provision of quality healthcare services to all citizens and, consequently, accomplishing the health‐related sustainable development goal agenda by 2030; the Department of Health should introduce additional techniques for engineering PHC with an emphasis on implementing primary preventive care to improve the implementation of PPC in PHC facilities.

**Reporting Methods:**

EQUATOR guidelines.

**Patient or Public Contribution:**

No Patient or Public Contribution.

## 1 Introduction and Background

Implementing primary health care (PHC) services has been playing a major role as an aspect that is prioritised by many low‐ and middle‐income countries. However, human, material and financial resource shortages facilitate poor progress towards achieving quality patient care. PHC facilities are a point of contact for community consumers who require preventative care, diagnosis and management of minor conditions (Mutshatshi and Munyai 2022). PHC facilities push for quality patient care through decent service delivery, affordable access, empowerment and sustainability, while many countries, including Africa, have adopted the PHC model. Even though the National Department of Health (NDoH) tries to make efforts in South Africa, quality healthcare provision remains a significant issue, especially in rural PHC facilities (Nesengani et al. 2025).

The study conducted in the Republic of Serbia reported an increase in chronic non‐communicable diseases (NCDs) such as cardiovascular diseases, malignant diseases and diabetes mellitus due to poor provision of Primary Preventive Care (PPC) services. Inadequate provision of PPC was associated with financial burdens on the healthcare system, the economic crisis and shortage of healthcare providers (Mitričević et al. 2021).

According to Muthelo et al. (2021), factors influencing poor implementation of PPC hold back the development of quality health services in all PHC facilities of South Africa in rural areas. Structured strategies through implementing PPC in the PHC system are known to strengthen health care services to achieve quality healthcare for the health professionals and community‐based consumers to reduce the incidence of disease burden in communicable diseases (CDs) and non‐communicable diseases (NCDs) at PHC facilities (Kwinika 2022). These strategies include accessibility of health care services to all community members of the public in all countries worldwide (Simon 2022). PHC professionals, the Department of Health, and the community are important in properly implementing PPC.

Various studies conducted worldwide explored and reported that poor provision of PPC was associated with a shortage of professional staff, a lack of skills in clinical practice, unavailability and inaccessibility of PHC facilities and unequal distribution of funds and material resources. All these led to a high risk of CDs and NCDs (Chan et al. 2021; Duda‐Sikuła and Kurpas 2023; Fufaa 2024). Similarly, poor implementation of PPC in PHC facilities of South Africa includes material resource factors such as speculums, glucometers and blood pressure machines; urine and pregnancy test strips; inadequate provision of preventive medications, including contraceptives, hypertensive medicines for pregnant women and immunisation vaccines (Simon 2022).

Other factors related to poor implementation of primary preventive care include human resource factors, such as a staff shortage in PHC facilities. These financial factors include poor allocations of funds, prioritisation of curative care over preventive care in PHC facilities, negative attitudes and beliefs of patients towards PPC (Simon 2022; Duda‐Sikuła and Kurpas 2023; Fufaa 2024). Furthermore, the rise in NCDs such as cardiovascular diseases, diabetes, cancers and chronic lung disease is responsible for 70% of global mortality over the past decade, with at least 75% occurring in Low‐Middle‐Income Countries (LMICs), which is the result of poor implementation of PPC in PHC facilities around the globe. Training of health care professionals and modification of regulations that guide health professionals can decrease the high rate of the burden of disease, as nurses will be well equipped with skills and knowledge to combat the alarming problem in the country (Endalamaw et al. 2024).

The study conducted by Ramovhoya and Nesengani (2022) further reveals that personnel shortages, heavy workloads and medication shortages impede effective care in implementing PPC. The South African National Disease Profile reported that in Limpopo Province, PHC facilities have the second highest disease burden percentages of NCDs and CDs at 53.5%, topped by Western Cape with 70.3%, which is more likely to be associated with poor implementation of PPC (Mckenzie et al. 2017). The researchers identified the gap in the implementation of PPC that could prevent CDs and NCDs and improve the quality of practice. Researchers utilised the interview guide to explore an in‐depth understanding and gain rich information on factors influencing the poor implementation of PPC in the PHC facilities in a selected Municipality.

## 2 Methodology

### 2.1 Study Design

The study adopted a qualitative approach using an exploratory, descriptive design to answer the research questions and achieve the study's objective. The researchers explored and obtained in‐depth information about factors influencing poor implementation of PPC in the PHC of a selected municipality. A thick description of participants' feelings and opinions was made to promote a deeper understanding of the phenomenon under study (Groove et al. 2015).

### 2.2 Study Setting

The study was conducted in the clinical setting of five selected PHC facilities of a selected municipality. Makhado PHC facilities provide health education, health awareness, health promotion, preventive care, maternity care and general patient care.

### 2.3 Study Population, Sample and Sampling

The study population consisted of registered professional nurses (males and females) who agreed to participate. Researchers used a non‐probability purposive sampling approach to select fifteen participants and five PHC facilities. The participants were selected based on their experience working in PHC facilities, and they were considered to provide rich, insightful data for the study, as an in‐depth understanding of factors contributing to poor implementation of PPC in PHC facilities is needed. Those were all registered professional nurses (males and females), aged 25–65 years, with 2 years of working experience in the PHC facilities. Participants who were available and agreed to participate in the study were recruited as they were deemed familiar with the PPC measures. The selection of PHC facilities was based on the types of services offered, their relevance and their potential impact on the study.

### 2.4 Data Collection Procedure

Researchers made appointments by visiting all selected Makhado PHC facilities' managers after receiving letters of permission from the Limpopo Provincial Government Director of Research and the DoH Vhembe District Chief Director to conduct the study. The interviews were conducted during late hours, around 16 h, to avoid service disruption because PHC facilities are less busy during these hours. The interviews took place in a natural setting, and the researchers ensured the environment was quiet, comfortable and conducive. The data collection process was done for 1 month. The first researcher was a male student pursuing a Master's degree, and the second researcher was the supervisor who attained a PhD and worked as a lecturer in that institution.

Before the interview commenced, the first researcher explained the study's purpose, objectives and significance to the participants to make them feel comfortable and at ease. Researchers provided participants with information leaflets to familiarise themselves with what was expected and participants signed a consent form. The researcher explained to the participants the use of the voice recorder, which they accepted. Interviews were conducted during participants' free time to ensure that the interviews did not interfere with their work; interviews were conducted in English. The researchers interviewed registered professional nurses who met the inclusion criteria and agreed to participate; none refused. However, researchers reached data saturation after interviewing 15 participants, and no additional new data was explored. Research interviews lasted between 45 and 60 min.

The researchers used a semi‐structured interview guide with questions formulated in English based on the research objectives. What factors influence the poor implementation of primary preventive care in primary health care facilities? What are the impacts of poor implementation of primary preventive care in primary healthcare facilities? That was followed by probing to get detailed, rich, comprehensive and in‐depth participants' data. Each interview session lasted about 30 to 45 min. Field notes were written immediately after the interview sessions to avoid missing some aspects of the data. Field notes refer to the information recorded by the researcher from observation during the interview sessions.

### 2.5 Data Processing and Analysis

The researcher used Tesch's eight processes for data analysis and examined the data (Cresswell 2016). The collected data was read and re‐read during this process to obtain a sense of all the information. A list of data was made available on cards, and editing was performed to make the gathered information retrievable and to discard unmanageable aspects. This process enabled the researcher to familiarise himself with the collected data, summarise the field notes and scrutinise the participants' words. Thereafter, the researcher developed codes into categories to enable an understanding of the participants' construction of reality. The final step of the analysis was to interpret and represent the data, give an overall description of the notes and analyse the data by organising it into manageable themes and sub‐themes.

### 2.6 Ethical Considerations

The researcher obtained an ethical approval certificate from the University research ethics committee and has a project number. Permission for the conduct of the study was received from the Provincial Research Committee Director, Limpopo Department of Health, and the District Executive Manager of Vhembe District to access selected PHC facilities of a selected municipality.

### 2.7 Recruitment of Subjects

Efforts to identify and recruit potential human research subjects should be designed to respect personal rights to privacy and confidentiality (Van den Brink and Koste 2015). The rights of participants were protected. A direct discussion to recruit subjects was used.

Written informed consent was obtained from participants before conducting the study. Participants were told that participation in the study is voluntary, and they have the right to terminate their participation at any stage of the project, despite their initial consent, without any adverse consequences. Using a voice recorder to capture the proceedings of the interview sessions was explained, and permission was obtained. Participants were informed of the field notes that would be taken, and their importance was clarified. Participants were assured of anonymity and confidentiality; they were not forced to participate in the study. It was made clear to participants that they were free to terminate their participation anytime they felt uncomfortable, and there would be no penalty. Participants were informed that in case of any emotional distress associated with the study, they would be referred to psychotherapy for counselling.

### 2.8 Trustworthiness

Principles for ensuring the trustworthiness of data were observed. Credibility was ensured through prolonged engagement, which occurred when the researcher visited participants, had information sessions with them, and made appointments during the preparatory phase to establish rapport. Transferability was ensured by selecting participants representing the entire population and presenting a thick methodology description. Dependability was ensured by sending the findings to the supervisors and co‐supervisors for an audit trail. Conformability was ensured by guaranteeing that the data supported the findings, conclusions and recommendations and that there was an internal agreement between the investigator's interpretation and the actual evidence.

## 3 Results

The study's results comprise the demographic profile of the participants and the findings from the interview conducted with 15 registered professional nurses regarding factors influencing poor implementation of PPC in PHC facilities.

### 3.1 Demographic Characteristic

The demographic characteristics of the registered professional nurses who participated in the study are as follows: Females constituted 87% (13) of the participants, and males constituted 13% (2). Regarding age, 7% (1) of the participants were in the age bracket of 25–29 years, 13% in the age bracket of 30–34 years, 27% (4) in the age bracket of 35–39 years and 53% (8) were 40 years and above. Regarding the working experience, 40% of the participants had 2–10 years of working experience and 60% had 11 years or more.

### 3.2 Presentation of the Findings

Two main themes indicating the contributory factors and the impact of poor implementation of PPC emerged, each with three themes. Eleven sub‐themes emerged and are supported by the quotes from the participants. These are presented in Table 1.

**TABLE 1:** Main themes, themes and sub‐themes reflecting poor implementation of primary preventive care.

Main theme	Themes	Sub‐themes
1. Participants' expression on factors contributing to poor implementation of primary preventive care	1.1. Financial management‐related factors	1.1.1. Poor allocation of funds for primary preventive care 1.1.2. Prioritisation of curative care at the expense of primary preventive care
	1.2. Factors related to resources	1.2.1. Shortage of human resources 1.2.2. Shortage of material resources
	1.3. Attitudes and beliefs of consumers towards services	1.3.1. Negative attitude of patients towards primary preventive care 1.3.2. Beliefs of patients towards primary preventive care
2. Participants' expressions regarding the impact of poor implementation of PPC	2.1. Personnel‐related impacts	2.1.1. Emotional suffering due to burnout among nurses 2.1.2. Attitude of nurses towards primary preventive care
	2.2. Support system	2.2.1. Poor working relationships among nurses, patients and the DoH 2.2.2. Poor support
	2.3. Increased burden of disease	2.3.1. High incidence of communicable and non‐communicable diseases

*Source:* Researchers' initiatives (2025).

### 3.3 Main Theme 1: Participant Expression on Factors Contributing to Poor Implementation of Primary Preventive Care

From the data collected, the majority of participants expressed various factors that influence the poor implementation of PPC in the PHC facilities of Makhado Municipality. From the two main themes, five themes and eleven sub‐themes that emerged are presented with supporting quotes:

#### 3.3.1 Theme 1.1: Financial Management–Related Factors

Effective implementation of PPC requires sufficient funds to maintain and improve the quality of life. Most participants expressed a need for adequate funds to achieve optimal health. Three subthemes emerged from this theme.

##### 3.3.1.1 Sub‐Theme 1.1.1: Poor Allocation of Funds on Primary Preventive Care

Most participants identified the poor allocation of funds on PPC as a barrier to the implementation of PPC in PHC facilities. The participants expressed that they lack medical devices for the effective implementation of PPC, and they blamed the DoH for not allocating enough funds to the PHC facilities.

Excerpts from the participant support this:

The personnel from our district offices always tell us that they do not have sufficient budget to meet all our needs. They usually complain that the budget allocated to us is enough! (P K, 26‐year‐old male)

Another participant said:

Since the outbreak of COVID‐19, many of the services were neglected by the Department because many funds were allocated for COVID‐19 services, as the focus was on it. Primary prevention was to depend on ineffective health education for most customers. (P E, 27‐year‐old female)

When there are insufficient funds, the implementation of PPC becomes ineffective as enough funds are needed to purchase sufficient diagnostic devices, preventive medications, supplies and hire enough healthcare personnel.

##### 3.3.1.2 Sub‐Theme 1.1.2: Prioritisation of Curative Care at the Expense of Primary Preventive Care

The research findings show that curative care is prioritised over PPC. Most participants identified this as a contributory factor to the poor implementation of PPC. Participants expressed that:

People in our community are more interested in the curative measures than the preventive care; I am saying this because mostly when we advise them to take the condoms from the clinic so that they engage in safe sex, they usually refuse, saying that they do not want to use the condoms, yet, they later come complaining of sexually‐ transmitted infections (STIs) including gonorrhea, syphilis and genital warts which could be prevented by using condoms without any cost. This shows that poor implementation of PPC is still a burden to clinics and the community. (P B, 42‐year‐old female)

Another participant said:

More curative medications and medical devices are readily available in our facilities, and the mere preventive medications and medical devices used in preventive care are always insufficient; for example, we always have medications for the management of Hypertension despite there being no calcium carbonate for pregnant women in antenatal care. Where are we going as a department if this is what we do to the patients? (P A, 35‐year‐old female)

Most participants concurred that preventive care is crucial and that the Department of Health and the South African people should now prioritise PPC over curative treatment. Findings about the Shortage of human and material resources are reported below:

#### 3.3.2 Theme 1.2: Factors Related to Resources

Resources play a significant role in delivering health care services, including PPC. Poor management of resources could lead to poor implementation of PPC. Two sub‐themes emerged from this theme.

##### 3.3.2.1 Sub‐Theme 1.2.1: Shortage of Human Resources

A lack of human resources was found to be a factor that influenced the poor implementation of PPC. Most participants emphasised that a scarcity of healthcare workers hampers their PHC facilities' inability to adopt PPC successfully.

One participant said:

Here in this facility, in each shift, we mostly have only two registered professional nurses; sometimes, if one happens to be sick, there will be only one registered professional nurse per shift. This is a huge shortage of staff, which influences poor implementation of PPC since many activities are not well implemented, such as giving patients health education, conducting campaigns, and rendering quality health care. (P O, 54‐year‐old female)

One participant expressed positive and said:

Even though some clinics have poor staffing, we have enough staff members because there are three registered professional nurses per shift. Every morning, we manage to educate our patients on health. We can even in‐service each other once weekly for the provision of quality health care. (P D, 34‐year‐old male)

##### 3.3.2.2 Sub‐Theme 1.2.2: Shortage of Material Resources

Lack of material resources has been a significant issue for several of Makhado Municipality's PHC facilities. Some of the medical equipment for diagnostics is still insufficient, and the shortage of these materials makes it difficult for PHC facilities to implement PPC. This is expressed by a participant below:

Another participant said:

We have challenges with equipment in our clinic; some we do have, and we turn to use them, but as often as we use them, they lose quality. Some we use and discard include speculums, needles, gloves, bandages, etc. The problem is that it takes time to deliver that equipment once they are finished, which takes us back to providing patients with poor PPC. (P C, 43‐year‐old male)

Another participant said:

In this facility, the biggest problem is the shortage of blood pressure monitoring machines, pregnancy tests, and glucometer strips. Those are the equipment that gives us challenges because every sick patient must be checked for blood pressure to rule out Hypertension and pregnancy in women who are not on birth control before we can help them. Still, without those diagnostic medical devices, making proper diagnoses becomes difficult for us. (P J, 61‐year‐old female)

Another participant said:

In our clinic, the most challenging thing is a Shortage of urine test strips because we often need them when women come for Ante‐natal visits to exclude Pregnancy Induced Hypertension (PIH) and urinary tract infections, but without them, it becomes a problem as we are experiencing a lot of maternal complication as we are failing to manage patients accordingly. Even the lack of glucometer strips is still a challenge because every patient has diabetes unless proven otherwise, so this also reduces our morale to work with passion and trust in ourselves due to those shortages. (P E, 27‐year‐old female)

Findings about the negative attitudes and beliefs of consumers towards PPC are reported below:

#### 3.3.3 Theme 1.3: Negative Attitudes and Beliefs for Consumers Towards Primary Preventive Care

Most participants reported that negative attitudes and beliefs of consumers towards PPC also contribute significantly to the poor implementation of PPC in PHC facilities. Two subthemes emerged.

##### 3.3.3.1 Sub‐Theme 1.3.1: Negative Attitude of Consumers Towards Primary Preventive Care

The participants expressed that the consumers display a negative attitude towards PPC.

This is supported by one participant who said:

I remember the other day this patient, who is a chronic patient, and we had to take blood for a better understanding and diagnosis of his problems, but the patient refused to allow blood to be taken, which left us with no choice but to protect the patient's rights, although it's a dilemma in our profession! The patient signed a refusal of treatment form reporting that his religion forbids the removal of tissues from their bodies. (PI, 31‐year‐old female)

Another participant said:

There are patients who are displaying a terrible attitude towards the vaccine for the Coronavirus (COVID‐19); this is defeating the effort of the DoH in trying to slow down the spread of the Coronavirus. (P M, 29‐year‐old female)

##### 3.3.3.2 Sub‐Theme 1.3.2: Beliefs of Consumers Towards Primary Preventive Care

Beliefs also play a significant role in the poor implementation of PPC. Some participants expressed their religious beliefs that hinder the implementation of PPC.

One participant said that:

A few patients have a good belief towards preventive care from our clinic because they adhere to the health education we give. Some patients have their own religious beliefs. One patient told us that contraceptives are not important, as the bible says they have to give birth to as many children as they can to fill the world. We have many women who have complicated pregnancies and deliveries because they are having many children without even spacing the births of their children. (P B, 42‐year‐old female)

### 3.4 Main Theme 2: Participants' Expression Regarding the Impact of Poor Implementation of Primary Preventive Care in Primary Healthcare Facilities

The research findings revealed various impacts related to the poor implementation of PPC in PHC facilities. Two themes and five sub‐themes emerged.

#### 3.4.1 Theme 2.1: Personnel‐Related Factors

Most participants expressed various personnel‐related factors associated with the poor implementation of PPC at PHC facilities. Four sub‐themes emerged.

##### 3.4.1.1 Sub‐Theme 2.1.1: Attitude of Nurses Towards Primary Preventive Care

The study's findings also demonstrate that some permit their disapproval of PPC and their pessimistic outlook to prevent them from carrying out their responsibilities as specified in their scope of practice. One participant said:

There are some nurses who facilitate the poor implementation of PPC because they take their beliefs to work and they somehow discourage the patients from practicing some preventive care as they believe that certain preventive measures are unnecessary or useless, like telling patients who want to terminate a pregnancy that is not a good thing to do as God does not like. These nurses have forgotten that their scope of practice is to give health education to the patient and allow the patient to make their own decision. (P D, 34‐year‐old male)

Another participant said:

To tell you the truth, there are some nurses who are so rude to the patients that they cause the patients not to come to the clinic for preventive care. Some nurses get mad at young girls who are below the age of 18 who come for contraceptives; they scold them and call them names, so this makes them stop coming to the clinic for contraceptives, and we end up having more teenage pregnancies. (P G, 36‐year‐old female)

That is supported by another participant who said:

I still remember this other nurse I worked with telling a patient from a foreign country that she must stop bringing her child to the clinic for immunization; she must go to her own country. This is not good because this woman is currently living here and is entitled to health care. So you see that this will also make patients from foreign countries who are currently living here stop attending for their children's immunisations, antenatal care, and other minor diseases they might be suffering from. This also put other community people in danger of some infections that could have been prevented. (P C, 43‐year‐old male)

##### 3.4.1.2 Sub‐Theme 2.1.2: Emotional Suffering due to Burnout Among Nurses

Emotional discomfort, especially among those who have 11 years of experience or more. Most of them (9) were exposed to heightened stress, burnout and absenteeism, exacerbated by the poor PPC implementation of PHC facilities. The participants reported that there have been inadequate diagnostic instruments, preventive drugs and supplies for more than 5 years, and that this compromises the implementation of PPC, including quality care.

This is a statement from a participant who said:

To tell you the truth, it is terrifying to help a patient with the COVID‐19 symptoms without the full protective gear. We have been working and attending to patients with those symptoms without the N95 mask. That causes us a lot of stress, and sometimes we miss ourselves from work because it is scary; we are humans, too. Working without all the necessary tools to help the patients in totality frustrates me, and this is one factor contributing to nurses absenting themselves from work. (P H, 32‐year‐old female)

That is further supported by another participant who said:

If the government fails to properly implement PPC for its workers, what about the patients? I refuse to attend to the patient without protecting myself, and yes, sometimes I end up not coming to work knowing that if I come, I will be at risk because I will be forced to work without proper Personal Protective Equipment (PPE). (P D, 34‐year‐old male)

#### 3.4.2 Theme 2.2: Support System

Most participants stated they required assistance from managers, supervisors and the health department. A lack of assistance demotivates nurses who apply for PPC. A subtheme surfaced.

##### 3.4.2.1 Sub‐Theme 2.2.1: Poor Working Relationship Between Nurses, Patients and the Department of Health

Most participants indicated that poor working relationships among the nurses, patients and the DoH negatively affect the implementation of PPC in the PHC facilities.

That is expressed by a participant who said:

If the DoH can start to have good relationships with the nurses in the PHC facilities, we could come up with solutions to most of our problems. If the DoH makes frequent support visits, they will know what we need. Some nurses get frustrated that the health personnel at the district and provincial levels do not regularly visit the facilities, but are always demanding quality patient care, while we need many missing equipment to achieve that. (P F, 53‐year‐old female)

Another participant said:

I believe in the spirit of Ubuntu, which is why I have a good relationship with my patients. It becomes easy for me to convince my patients to do certain health screenings, and they understand me better when I give them health education. (P K, 26‐year‐old male)

The research findings revealed the factors associated with poor implementation of PPC: increased disease burden, increased expenditure for the health care department, increased workload in secondary‐health care facilities, absenteeism and burnout and low work performance.

#### 3.4.3 Theme 3: Increased Disease Burden

Participants expressed an increase in disease burden as one of the impacts associated with poor implementation of PPC in the PHC facilities.

That is supported by a participant who said:

The impact of poor implementation of preventive care is detrimental to the health status of the entire South African population, and it adds more pressure to the DoH and its employees. All this causes an increase in nurses' workload, leading to an increase in disease burden. (P D, 34‐year‐old male)

Another participant also said:

The number of patients who are on anti‐retroviral treatment (ART) for HIV is too high, and it is shocking. This can only mean one thing, which is that people are not practising safe sex. They are not using condoms, and this is putting a strain on the DoH. (P E, 27‐year‐old female)

One other participant said:

If people were so positive about the preventive care measures, not only concerning COVID‐19 but all infections, we would have significantly reduced the rate of coronavirus infection in our country. However, people are not adhering to the preventive measures, thus causing a rapid rise in the COVID‐19 infections and disease burden. (P N, 49‐year‐old female)

## 4 Discussion of the Findings

The Department of Health in South Africa tried to uplift the health system by implementing the National Health Insurance (NHI) for quality health services at low cost to all patient PHC facilities; however, other countries in Africa adopted innovative PHC programmes to implement PPC, such as Ethiopia's Health Extension Program, which engaged community health workers (CHWs) to deliver essential services by visiting patients at home and making follow‐ups to maintain adherence, and Rwanda's community‐based health insurance scheme to improve accessibility and affordability of health services. Despite these advancements, the implementation of PPC in PHC facilities across Africa remains a huge problem, with a lot of countries going through significant challenges, such as resource constraints, workforce shortages, inadequate infrastructure and weak supply chains, all of which hinder the full implementation of PPC and awareness of PHC goals (Nesengani et al. 2025).

Through many literature reviews and emerging themes and sub‐themes, the study revealed that poor implementation of PPC faced by PHC facilities of Makhado is also a challenge faced by other African countries. Different factors related to the poor implementation of PPC in PHC facilities were explored, including human and material resource factors, financial factors, personnel‐related factors the attitudes and beliefs of healthcare consumers expressed during the data collection process. The study revealed that these factors can be prevented if proper measures are taken by the Health Department, healthcare personnel and the public to improve quality of life.

Through data collections, some of the participants under financial factors mentioned that they still face shortages of supplies such as gloves, needles and many more health appliances for rendering essential services. Patients worry about their health by improving their health, and some lose hope that quality health care exists. Some facilities complained about shortages of medications such as vaccines, which delay children from being vaccinated at the right time. Some turn to forfeit the vaccination period and become exposed to deadly diseases, such as measles and polio, and the Bacillus Calmette‐Guérin vaccine (BCG) has a short period. The revelation of this study clearly showed that the primary people of a selected Municipality are facing challenges; however, some provinces of South Africa and Africa are also going through these challenges. Limpopo province is one of the provinces where most people live in deep rural areas in poverty. Mobile health care services are rarely rendered due to poor road structure and slippery conditions during the summer seasons. Consumers can be serviced once a month, sometimes not. That also creates a huge burden when delivering and providing PPC services. In areas where PHC facilities are available, most medications require storage temperatures, especially coldness and freezing. Still, some of the facilities are having challenges with backup generators; some do not have them at all, so when electricity is off, as South Africa is shadowed by load shedding, the effectiveness of medications is reduced and can lead to resistance, especially antibiotics (Chinawa 2015; Kwaskebe et al. 2022). The shortage of human resources in the PHC facilities was associated with the shortage of primary healthcare professionals such as nurses, doctors, clerks, poor job security and low salary levels. In the PHC facilities, it is revealed that in a shift, nurses on duty should be at least three registered professional nurses, one enrolled nurse and one enrolled nurse assistant working day shift and at night shift it is two registered professional nurses, one enrolled nurse, one enrolled nurse assistant and one cleaner; both shifts include three security personnel per shift, as amended by the Department of Health Limpopo in 2016.

Despite these amendments by the Limpopo Department of Health, the staff shortage is still a big challenge in many PHC facilities in a selected Municipality. In some facilities, they complained about this problem, as this strains them because of the large number of health consumers they serve. This causes long waiting times; nurses are unable to give health education to patients in the morning, to perform in‐service training and to conduct data quality meetings, which led to poor implementation of PPC and poor health delivery to the community. Professional nurses multitask and even work out of their scope, which indicates that in PHC facilities, there is a shortage of staff. Some of the PHC facilities operate without pharmacist assistance, as medications need to be dispensed on a daily basis; yet it's nurses who do all tasks, even retrieving files on holidays and weekends; it's for them to do. That negatively affects PPC delivery because of the workload on the limited number of healthcare professionals in PHC facilities, and it exposes healthcare workers to incidents involving patients, which may lead to litigations and suspensions (Kwaskebe et al. 2022). Therefore, it is essential to allocate enough funds for better provision of medications at the PHC facilities to improve the implementation of PPC in PHC facilities to benefit the population's health and healthcare systems. Nurses require non‐nursing tasks; if their burden grows, they may do less in some of these responsibilities. Therefore, the facility will probably focus on reducing the length of the lines rather than providing health education to enhance PPC implementation.

Misallocation of funds and increased expenditure on curative rather than preventive services hindered the implementation of PPC in PHC facilities of Makhado and other provinces in South Africa. One of the DoH's most significant issues is a lack of funding. If this issue is resolved, the budget will grow and provide sufficient resources for PPC implementation in PHC facilities. Even though preventative care services don't cost much, money is still needed to buy the necessary materials. A possible contributing factor to the lack of diagnostic medical equipment, such as specula, glucometer machines and pregnancy test strips, is the inadequate funding provided to PHC facilities. In addition, the PHC facilities lack healthcare awareness and an insufficient provision of medications such as folic acid, calcium, ferrous sulphate and contraceptives, among other preventive drugs.

According to a study by Rubio‐Valera et al. (2015) conducted in the USA, funding and other resources may act as both common obstacles and enablers for adopting PPC in PHC systems. They say inadequate funding allocation and a lack of supplies and equipment contribute to the subpar PPC implementation at PHC institutions. Most PHC facilities continue to lament the absence of diagnostic medical equipment and supplies, which impede the implementation of PPC in Makhado's PHC facilities. Without these resources, nurses in PHC facilities cannot accurately diagnose patients, resulting in mishandling and even legal action taken against the nurses (Ntoimo et al. 2019). According to a related study, staffing shortages accounted for almost 78% of patient wait times, leading to complaints and mistrust of nurses and poor PPC implementation in PHC facilities (Burger and Christian 2018; Mutshatshi and Munyai 2022).

Programs that raise public awareness of healthcare issues are essential to the PPC's implementation because they help disseminate health education throughout the community. However, most PHC facilities continue to struggle greatly to carry out various awareness campaigns, which increases the burden of sickness and leads to complaints from health professionals. Healthcare awareness programs, such as cancer and immunisation campaigns, are not adequately carried out since the Department of Health does not provide financial support due to an insufficient budget. The Department of Health (DoH) focused solely on COVID‐19 during the pandemic, disregarding other issues, including HIV, TB and vaccination awareness.

Despite the advantages of preventative care, a comparable study conducted in the United States of America in 2016 discovered that the average amount spent on public health and prevention was just over 3% of public spending, while the average amount spent on curative care was 57% (Organisation for Economic Cooperation and Development 2016). In other studies, it is reported that a lot of funds are allocated to urban PHC facilities for types of equipment, supplies, staffing and little is allocated to rural PHC facilities. This results in an increase of 33% NCD and 29% CD in rural health facilities due to insufficient materials and increased workload among available health professionals (Getachew et al. 2017; Ekenna et al. 2020). Okonofua et al. (2018) and Kanyangarara et al. (2018) reported similarly that maternal healthcare services were also compromised by poor implementation of PPC in PHC facilities in Nigeria and Ethiopia, respectively, and that is a significant concern.

Similar results were found in a Canadian study by White (2015), which showed that the Health Department continues to provide insufficient material resources to most Canadian healthcare facilities to implement PPC. As a result, the nation continues to experience inadequately executed preventative care programmes, raising the incidence of disease in Canada. Because nurses prefer to work in well‐resourced medical facilities, staffing shortages arise with limited material resources, contributing to subpar PPC implementation in PHC facilities. The PHC facilities that have more material resources also attract more human resources, as it is widely known that one of the leading causes of migration of nurses from one country to another is the shortage of material resources (Kanyangarara et al. 2018).

Everyone in South Africa can practise their religion as they see fit. Still, as nurses have sworn to provide complete care and to prioritise their patients, their personal beliefs should not interfere with their nursing obligations (Simon 2022). Because nurses are required to carry out all nursing duties following their scope of practice, it is unacceptable for them to use their personal beliefs to justify failing to provide patients with PPC. Patients seeking PPC have a right to receive complete medical care without fear of judgement. Similarly, patients exhibit unfavourable attitudes towards the PPC, including ignorance and cultural beliefs. That makes it more difficult for the DoH to reduce the high rate of illnesses and promote universal health. The way nurses treat patients impacts not just the patients but also the Health Department as a whole. That is because a higher illness burden indicates inadequate PPC implementation. Similar research using the ecological model framework by Rubio‐Valera et al. (2015) found that attitudes and beliefs play a significant role in applying PPC and can act as both a facilitator and a barrier to it. The authors also noted that the application of PPC at PHC facilities may be hampered by limited awareness of the intervention and a lack of motivation. The way nurses treat patients impacts not just the patients but also the Health Department as a whole. A higher illness burden indicates inadequate PPC implementation in PHC facilities.

The DoH, patients and nurses have a positive connection that affects how PPC is implemented at PHC institutions. Adequate PHC is delivered with the support of positive and trustworthy interprofessional relationships fostered by bidirectional communication, ample opportunities for staff to discuss important issues and their input on obstacles before and during PPC implementation. Nonetheless, participants in this study expressed having strained relationships, which hinder teamwork and push some patients to stop receiving treatment because they feel unwelcome in the PHC facility. According to a study by Rubio‐Valera et al. (2015), some nurses tend to be absent from work due to boredom and worry sparked by patients' behaviours. Similarly, patients who quit going to PHC facilities may do so out of concern for the nurses' reactions to personal difficulties. According to a similar study done in South Africa by Massyn et al. (2015), most patients at PHC facilities are unwilling to seek preventive care services because they have a bad rapport with the nurses. As a result, they fail to seek PPC from healthcare professionals, increasing the disease burden. Similar findings were also observed by Burger and Christian (2018), who found that 38% of patients avoid the closest PHC clinics because they believe the nurses are indifferent and unkind.

The ideal situation of all clinics being visited by a doctor at least once a week was not met in most districts of the province, especially those in rural settings. Although the province had the country's highest public sector vacancy rate at 86%, it is still struggling with the shortages of human and material resources, financial burdens and poor implementation of PPC in PHC facilities (Massyn et al. 2015). That is a significant concern as consumers of healthcare services still do not have faith in the effectiveness of modern medical care services, and they usually have negative attitudes towards PPC services offered in the PHC facilities. Most still believe in traditional African medicine.

## 5 Strengths and Limitations of the Study

The study could significantly contribute to realising universal healthcare and accomplishing the health‐related Sustainable Development Goal agenda by 2030. The Department of Health should introduce additional techniques for engineering PHC, emphasising PPC implementation. Although the research method complied with the study's standards, the study was done in a particular setting with a relatively small sample size. Because of this, the study's conclusions cannot be applied generally, but they can serve as a basis for future research on related topi**cs**.

## 6 Conclusion

Several factors have contributed to the inadequate implementation of PPC in PHC facilities. These include the financial strain, scarcity of people and material resources and the attitudes of healthcare customers and nurses. All of these have contributed to increased rates of CDs and NCDs in the Makhado municipality, as well as mental stress and strained relationships between nurses, the health department and patients. To achieve universal health, the DoH must act quickly to address the issue of poor PPC implementation at the PHC facilities of Makhado Municipality.

## 7 Recommendations of the Study

To successfully implement PPC, the Department of Health must advocate for hiring all qualified registered professional nurses to address the nursing staffing deficit. The increased budget for preventive care could curb the poor implementation of Primary Preventive Care services in primary healthcare facilities. All nurses need in‐service training on professional behaviour and nurse–patient interaction. That will help them understand how the Department of Health expects them to act in the workplace and prevent bad attitudes.

## 8 Implications for the Profession and Patient Care

Sufficient human and material resources could improve the implementation of primary preventive healthcare services, promoting the health outcomes of individuals, families, groups and the community. There would be a proper distribution of resources to meet the needs of people of all age groups. There would be a reduction in the mortality and morbidity rates due to the decline of non‐communicable and communicable diseases. Most professional nurses might be motivated and have high morale that could synergise their expertise effectively in providing health promotion and disease prevention through patient education. Patients with multiple comorbidities and chronic health conditions would have equal access to primary healthcare facilities free of charge in South Africa.

## Author Contributions

Bongani Kwinika conducted the study as a requirement for a Master's degree at the University of Venda and drafted the initial manuscript. Seani Adrinah Mulondo was the supervisor who reviewed, revised and finalised the manuscript, Both authors agreed to the final version of the manuscript for publication.

## Ethics Statement

This study received ethical approval from the University's Higher Degrees Committee (approval SHS/20/PDC/43/2021) on November 11, 2020.

## Conflicts of Interest

The authors declare no conflicts of interest.

## Data Availability

The datasets analysed during the current study are available through the corresponding author on reasonable request.
